# Therapeutic Approaches for the Management of Trigeminal Autonomic Cephalalgias

**DOI:** 10.1007/s13311-018-0618-3

**Published:** 2018-03-07

**Authors:** Diana Y. Wei, Rigmor H. Jensen

**Affiliations:** 1 Headache Group, Department of Basic and Clinical Neuroscience, Institute of Psychiatry, Psychology and Neuroscience, King’s College London, London, UK; 2 Danish Headache Centre, Department of Neurology, Rigshospitalet-Glostrup, University of Copenhagen, Copenhagen, Denmark

**Keywords:** Trigeminal autonomic cephalalgia, Cluster headache, Hemicrania continua, Paroxysmal hemicrania, SUNCT/SUNA

## Abstract

Trigeminal autonomic cephalalgia (TAC) encompasses 4 unique primary headache types: cluster headache, paroxysmal hemicrania, hemicrania continua, and short-lasting unilateral neuralgiform headache attacks with conjunctival injection and tearing and short-lasting unilateral neuralgiform headache attacks with cranial autonomic symptoms. They are grouped on the basis of their shared clinical features of unilateral headache of varying durations and ipsilateral cranial autonomic symptoms. The shared clinical features reflect the underlying activation of the trigeminal–autonomic reflex. The treatment for TACs has been limited and not specific to the underlying pathogenesis. There is a proportion of patients who are refractory or intolerant to the current standard medical treatment. From instrumental bench work research and neuroimaging studies, there are new therapeutic targets identified in TACs. Treatment has become more targeted and aimed towards the pathogenesis of the conditions. The therapeutic targets range from the macroscopic and structural level down to the molecular and receptor level. The structural targets for surgical and noninvasive neuromodulation include central neuromodulation targets: posterior hypothalamus and, high cervical nerves, and peripheral neuromodulation targets: occipital nerves, sphenopalatine ganglion, and vagus nerve. In this review, we will also discuss the neuropeptide and molecular targets, in particular, calcitonin gene-related peptide, somatostatin, transient receptor potential vanilloid-1 receptor, nitric oxide, melatonin, orexin, pituitary adenylate cyclase-activating polypeptide, and glutamate.

**Electronic supplementary material:**

The online version of this article (10.1007/s13311-018-0618-3) contains supplementary material, which is available to authorized users.

## Introduction

**Fig. 1:**
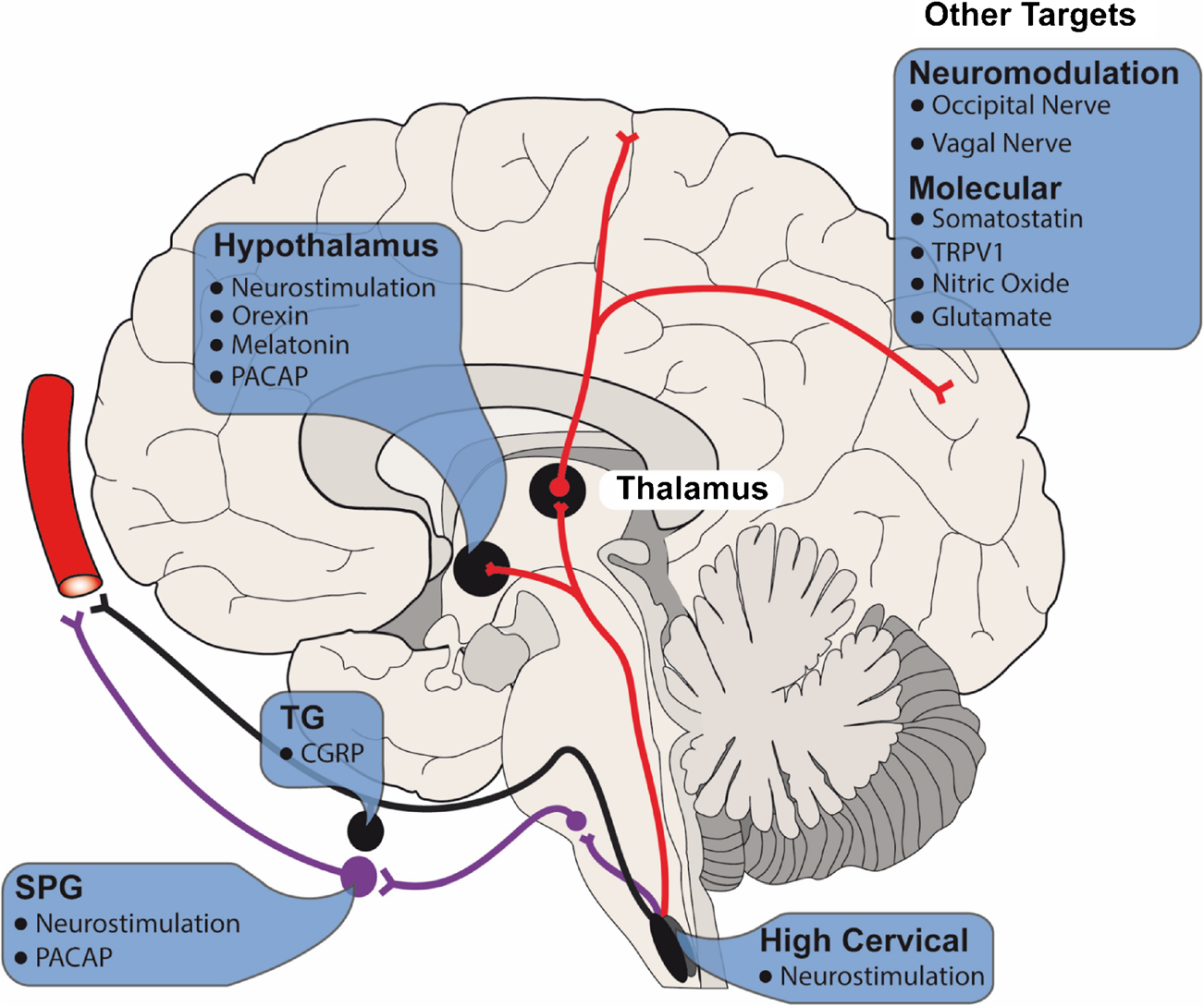
Overview of the therapeutic targets for trigeminal autonomic cephalalgia conditions, identifying both molecular targets as well as the structural targets for neurostimulation and neuromodulation as outlined in this review. TG = trigeminal ganglion; CGRP = calcitonin gene-related peptide; SPG = sphenopalatine ganglion; PACAP = pituitary adenylate cyclase polypeptide; TRPV1 = transient receptor vanilloid

Trigeminal autonomic cephalalgia (TAC) encompasses 4 primary headache disorders that are characterized by their shared unique features. TAC is a term coined by Goadsby and Lipton in 1997 [1], in which they described the 4 conditions with cranial autonomic features as a separate entity to short-lasting headaches without cranial autonomic features. The initial conditions included were: cluster headache (CH), paroxysmal hemicrania (PH), short-lasting unilateral neuralgiform headache attacks with conjunctival injection and tearing (SUNCT)/short-lasting unilateral neuralgiform headache attacks with cranial autonomic symptoms (SUNA). Hemicrania continua (HC) officially appeared in the International Classification of Headache Disorders version 2 (ICHD 2) in 2004 [2]. Each condition presents with severe unilateral pain in the trigeminal nerve distribution of varying durations and ipsilateral cranial autonomic symptoms (Table 1). Their shared clinical features are the basis of this classification and gives rise to the underlying pathophysiology which involves the trigeminovascular reflex and the trigeminal–autonomic reflex. In this review, we will describe each TAC condition and discuss the neurotherapeutic targets, starting with structural targets for neuromodulation followed by molecular targets for future therapies (Fig. 1).

**Table 1:** Overview of the features of each trigeminal autonomic cephalalgia

Features	Cluster headache	Paroxysmal hemicrania	Hemicrania continua	SUNCT/SUNA
Gender ratio (male to female)	3:1	1:1	1:2	1.5:1
Pain quality	Sharp/stab/throb	Sharp/stab/throb	Baseline—dull pain. During worsenings can be sharp/throb	Sharp/stab/throb
Pain severity	Very severe	Very severe	Baseline—mild to moderate. Worsenings—moderate to severe	Severe
Distribution of maximal pain	V1 > C2 > V2 > V3	V1 > C2 > V2 > V3	V1 > C2 > V2 > V3	V1 > C2 > V2 > V3
Attacks per day	1-8	1-40	Daily in 50%	1-100
Attack duration	15-180 min	2-30 min	30 min to 3 days	1-10 min
Cranial autonomic features	Prominent and ipsilateral to pain	Prominent and ipsilateral to pain	Present during worsenings and can be bilateral	Prominent and ipsilateral to pain
Restlessness during attack	95%	80%	69%	65%
Circadian periodicity	Present	Absent	Absent	Absent
Triggers				
Alcohol	+++	+	+	−
Nitroglycerin	+++	+	−	−
Cutaneous	−	−	−	+++
Associated migraine features				
Nausea	50%	40%	53%	10%
Photophobia	56%	65%	74%	25%
Phonophobia	43%	65%	79%	25%
Treatment response				
Oxygen	Yes	No	No	No
Sumatriptan injection	Yes	Partial	Partial	No
Indomethacin	No	Yes	Yes	No

SUNCT = short-lasting unilateral neuralgiform headache attacks with conjunctival injection and tearing, SUNA = short-lasting unilateral neuralgiform headache attacks with cranial autonomic symptoms

### Cluster Headache

Cluster headache is the most common TAC and is well characterized by attacks of severe unilateral orbital, supraorbital, and/or temporal pain lasting 15 to 180 min when untreated, according to the International Classification of Headache Disorders 3 (ICHD 3) [3]. It affects approximately 0.1% of the population. The pain is often compared to and felt worse than that of fractured bones, renal colic, and child birth [4]. Each painful attack is accompanied by prominent ipsilateral cranial autonomic symptoms, which arise from parasympathetic overdrive: ipsilateral lacrimation, conjunctival redness, periorbital swelling, nasal congestion, rhinorrhea, aural discomfort, or sympathetic inhibition: ptosis and miosis. During the attack, patients have an intense sense of restlessness and agitation and most prefer to pace, rock, and press hard into the side of their face of the attack [5]. The attacks can occur once every other day to up to 8 times a day, with a circadian pattern, whereby attacks often occur at the same time each day and there is a tendency for nocturnal attacks. There is also a predilection for a circannual pattern, with an increased likelihood for attacks in spring and autumn [6]. Patients with episodic cluster headache have a cluster of attacks followed by a period of remission between attacks for more than 3 months without any preventive treatment, and chronic cluster headache patients have an absence of a remission period or remissions last less than 3 months for at least 1 year [3].

The unique clinical features of cluster headache in its circannual and circadian tendency [6, 7], as well the neurohormonal changes in testosterone [8–10], cortisol [11], and melatonin [12–15], support the hypothalamus as key player in the pathogenesis.

### Paroxysmal Hemicrania

Paroxysmal hemicrania (PH) is a rare TAC characterized by attacks that are shorter in duration than CH and with more attacks in a day. PH attacks are unilateral in the distribution of the trigeminal nerve and last from 2 minutes to half an hour. Attacks can recur up to 40 times a day with a mean of 11 attacks a day [16]. Patients can have photophobia and phonophobia lateralized to the side of the pain. The attacks are mostly spontaneous; however, in 10% of patients, the attacks can be triggered by head turning [17]. The attacks do not tend to occur at night, as cluster headache attacks do. A distinguishing feature is that PH is indomethacin sensitive compared to cluster headache [16, 18].

Episodic PH occurs in 35% of patients and is defined when there are remission periods lasting 3 months or longer, whereas in chronic PH, either there is the absence of a remission period or remissions last less than 3 months, for at least 1 year [3].

### Hemicrania Continua

Hemicrania continua is a continuous strictly unilateral headache, whereby the severity of the headache waxes and wanes with periods of worsening, without complete resolution. There can be associated ipsilateral cranial autonomic features during the worsenings; however, the features can be bilateral [19]. There can be associated nausea, photophobia, and phonophobia. Similar to PH, hemicrania continua is responsive to adequate doses of indomethacin [19].

### SUNCT/SUNA

SUNCT and SUNA are short-lasting unilateral attacks of pain, presenting typically in the V1 region of the trigeminal nerve. The attacks present in 3 types: single stab, a group of stabs, or in a saw-tooth pattern [20]. The attacks last from 1 to 600 seconds and occur multiple times a day, with a tendency of the frequency to be in the hundreds. Attacks can be triggered by a cutaneous trigger, such as touch, chewing, and brushing teeth. There is an absence of a refractory period to retriggering; in a recent cohort study, less than 5% of SUNCT/SUNA patients had a refractory period [21]. Each attack is associated with ipsilateral cranial autonomic symptoms. In SUNCT, there is conjunctival injection and tearing, whereas in SUNA, there is only one or neither of conjunctival injection and lacrimation [3].

## Pathogenesis

Our understanding of the pathogenesis for TACs has come from key bench work [22, 23] and functional neuroimaging studies. The unifying mechanism for TACs is the role of the trigeminal–autonomic reflex with parasympathetic activation [1]. The trigeminal–autonomic reflex is a reflex pathway that consists of a brainstem connection between the trigeminal nerve and facial cranial nerve parasympathetic outflow via the superior salivatory nucleus (SSN) and sphenopalatine ganglion (SPG). The postganglionic parasympathetic neurons contain nitric oxide synthase [24], vasoactive intestinal polypeptide (VIP) [25], and pituitary adenylate cyclase-activating polypeptide (PACAP) [26].

From functional neuroimaging studies, it has been shown that the hypothalamus is activated ipsilateral to the pain in cluster headache [27–30], contralateral in paroxysmal hemicrania [31], ipsilateral [32] and bilateral [33] hypothalamic activation in SUNCT, and contralateral in hemicrania continua [34]. There is further evidence to support the hypothalamic involvement from deep brain stimulation targeting the posterior hypothalamus in cluster headache [35–37] as well as changes in testosterone [8–10], cortisol [11], melatonin [12–15, 38–40], and orexin [41, 42] in cluster headache [43].

Management and treatment for TACs thus far have been limited and have not been targeted towards the mechanisms of the underlying pathophysiology. Recent advances in our understanding of TAC pathophysiology have allowed the development of neuromodulation therapies and pharmacological treatments targeting mechanisms known to be important in the disorder, thereby leading to better and more efficacious management of patients.

## Central Neuromodulation Targets

### Hypothalamus

Neuroimaging findings have demonstrated the posterior hypothalamus as a key area of activation during cluster headache attacks [27]. Furthermore, a voxel-based morphometry study demonstrated increased neuronal density in the posterior inferior hypothalamic gray matter outside the bout phase of cluster headache patients [44]. In 2000, Leone and colleagues [35] were the first to use stereotactic deep brain stimulation to target the ipsilateral hypothalamus in a drug-resistant chronic cluster headache patient; since then, at least 69 refractory chronic cluster headache patients have undergone this procedure [45]. From the cumulative data of 108 cluster headache attacks treated in 16 patients, it was effective in reducing the acute attack pain intensity by more than 50% in 23%, but it was not effective as an acute treatment, only 16% aborted the attack within 20 min stimulation [46]. A long-term follow-up (median, 8.7 years; range, 6–12 years) of 17 refractory chronic cluster headache patients showed an overall beneficial frequency response rate in 71% of the 17 patients, whereby 6 patients were almost completely pain free and 6 patients experienced long periods of remission, with the pattern of the headaches becoming episodic. It should be noted that there is often a significant delay between the initial use of the DBS and the clinical effect [37, 47]. The pain-free state has been shown to continue even after the stimulators had been off; this was found in 5 patients after years of continuous stimulation and then switching off for a median of 3 years [48]. This implies that chronic hypothalamic stimulation can modulate disease course. In another attempt to test the efficacy, a randomized controlled small study was initiated and there was no difference in the attack frequency with sham *versus* verum stimulation, but the randomization period lasted only 1 month [49]. Inherently, a proper long-lasting randomized control study is considered to be unethical and complicated to conduct.

One challenge to deep brain stimulation is accuracy and localizing the stimulation site. To date, there is no consensus whether it is the posterior hypothalamus or anterior periventricular gray matter [48, 50].

Deep brain stimulation is not risk free and the most serious side effect is fatal intracerebral hemorrhage; this occurred in 1 patient who died after developing an intracerebral hemorrhage [37]. Other side effects include visual disturbances, especially diplopia; this has been noted when the amplitude increased too rapidly but the diplopia resolves within a few minutes to days [51]. There has been a case of a medically refractory chronic cluster headache patient who developed recurrent paroxysms of sneezing soon after deep brain stimulation of the posterior hypothalamus was started [52].

Chabardes and colleagues [53] conducted a pilot study using less invasive approaches to reach the posterior hypothalamus to minimize side effects*.* They used an endoventricular approach to perform posterior hypothalamus deep brain stimulation using a MRI brain and contrast ventriculography. Seven chronic cluster headache patients were enrolled in this pilot study, with encouraging clinical outcomes. Initially, the procedure was attempted in 2 patients under local anesthesia; however, they suffered from vomiting during the insertion of the lead into the third ventricle. Therefore, the subsequent 5 cases were performed under general anesthesia. The clinical outcome at 12 months was encouraging; 3 patients had gone into clinical remission, 3 patients had gone into subtotal remission, and 1 patient, who had no response for the first 3 and 6 months, continued to have attacks but these attacks were reduced from the baseline amount. They did not see any intraparenchymal hemorrhages; however, there was a patient that required a repositioning of the electrode that had spontaneously moved. The side effects from stimulation included ipsilateral autonomic symptoms and “trembling vision” attributed to the stimulation of the upper brainstem regions around the pedunculopontine nucleus area.

In other TACs, there have been 3 drug-resistant SUNCT patients where deep brain stimulation has been implanted in the ipsilateral posterior hypothalamus with improvement [54–56]. In 1 patient, deep brain stimulation resulted in long-lasting pain relief without oral medications [54]; in another patient, there was an 80% reduction in daily frequency of attacks at 1 year after implantation [55]; and for the third patient, there was a decrease in attack frequency initially; however, the patient was not able to reduce the oral medications [56].

In a PH patient intolerant to medication, the ipsilateral posterior hypothalamus was implanted and this was effective in controlling acute attacks [57]. From neuroimaging, the posterior hypothalamic activation in PH was found to be contralateral to the side of attacks [31].

### High Cervical Nerves

High cervical spinal cord stimulation was first used in cluster headache in 2004 [58] and since then has been performed in 7 medically refractory cluster headache patients at low frequency [59]. The results showed that there was an immediate improvement in pain as well as a neuromodulation effect with reduced attack frequency, duration, and intensity. The disadvantage is that there is a high number of revision surgeries required due to electrode breakage and lead revisions. Some patients describe paresthesia induced by certain head movements. High-frequency spinal cord stimulation (HF10 SCS) targets stimulation at C2 to C3 levels and was tried in medication-refractory headache disorders, 2 patients with chronic SUNA and 1 patient with chronic cluster headache [60]. The authors reported the patients having an almost immediate therapeutic effect, but long-term observations are still required. It is postulated that by high cervical low-frequency stimulation it reaches the trigeminal cervical complex (TCC) and generates neuromodulation, but further evidence and randomized control studies are needed. The TCC is an important part of the trigeminal–autonomic reflex and transmits to the central pain centers.

## Peripheral Neuromodulation Targets

### Occipital Nerves

The greater occipital nerve emerges from the C2 spinal root, and near the occipital bone, the nerve may divide into branches supplying the posterior aspect of the head and vertex cutaneously. In rats, stimulation of the greater occipital nerve caused increased central excitability of the dural afferent in rats [61]. In cat studies, we know that stimulation of the greater occipital nerves increases metabolic activation in the cervical spinal region and the trigeminal nucleus caudalis in the brainstem [62]. The studies suggested that there was a 2-way connection between the upper cervical segments and the trigeminal branches via the trigeminal nucleus caudalis. The C1 to C3 cervical dorsal horns and the trigeminal nucleus caudalis form the TCC.

Greater occipital nerve (GON) region injections with corticosteroids and local anesthetics can provide a rapid benefit for patients and have been beneficial as a bridging therapy in cluster headache. Several studies have looked at the efficacy of greater occipital nerve blocks in cluster headache [63–67], the most recent being a prospective observational study, using 10 mg triamcinolone in 1 mL sodium chloride and 1 mL bupivacaine 0.5% to infiltrate both the greater and lesser occipital nerves [68]. They found more than 80% of patients had complete or partial response [68]. The methods vary between studies, with different constituents used in the injection and some studies using serial and/or bilateral injections; therefore, the results are not directly comparable and show a range of duration of benefit. In one study with 83 chronic cluster headache patients, they showed for a single injection to the greater occipital nerve using 80 mg methylprednisolone and 2 mL of 2% lidocaine, the median duration of benefit was 21 days, with the main side effects of lightheadedness, neck stiffness, and continuous pain at the site of injection [67]. A rare complication of permanent alopecia and cutaneous atrophy at the injection site has been noted [69].

Greater occipital nerve injections have also been used in a handful of the other TACs as an interim treatment. In a review by Afridi and colleagues [64], 3 SUNCT patients who had GON injections, there was 1 with complete response. Furthermore, Porta-Etessam and colleagues [70] showed the beneficial effects of GON injection in an 84-year-old woman with SUNCT, and presented it as a useful alternative treatment considering the potential risks of intravenous lidocaine in her case. More recently, Weng and colleagues [21] reported GON injections were beneficial in 50% (6 out of 12 cases) of SUNCT patients and 75% (3 out of 4 cases) of SUNA patients. In chronic PH, one case series of 6 patients did not observe any beneficial effect from lidocaine GON injections [71]. In a further case series, out of the 3 patients with chronic PH, 1 had a complete response to the GON injection [64]. Rossi and colleagues [72] reported a patient with indomethacin-responsive episodic PH, who responded to repeated lidocaine and methylprednisolone GON injections; interestingly, there was no clinical response when the patient was injected with a GON injection containing only lidocaine. In a case series of 7 HC patients, GON injections containing lidocaine were not helpful, but they found that there was some response to supraorbital injections [71]. In a separate case series of 7 HC patients, 1 had a complete response and 5 had partial responses [64].

Occipital nerve stimulation (ONS) was first used in medically refractory occipital neuralgia by Weiner and Reed [73], the pioneers of ONS, in 1991. Given the benefit from occipital nerve block using steroid and local anesthetic in cluster headache patients and the use of occipital nerve stimulators in migraine patients [74], ONS was used in medically refractory chronic cluster headache patients. It was tried in 2 TAC patients in 2006, 1 chronic cluster headache patient, and 1 hemicrania continua patient [75], with beneficial effects on headache frequency, duration, and intensity and an interesting observation that both patients still noted episodes of cranial autonomic features in the absence of the pain. Following on from this, there was a prospective pilot study of ONS in 8 medically refractory chronic cluster headache patients, with encouraging results [76]. They observed a delay of 2 months or more between implantation and significant clinical improvement.

Burns and colleagues [77] presented data from an open-label study of ONS in 8 cluster headache patients and showed that ONS was well tolerated with a lesser side effect profile than the more invasive deep brain stimulation. In the initial cases with unilateral stimulation inserted, the patients noticed side-swapping of their attacks, so the subsequent stimulators inserted were bilateral. They observed an interesting feature noted by patients with positive clinical effect, namely there was a pleasant paresthesia when the stimulators were turned on. The complications of ONS in this study included excessive pain following the operation and shock-like sensations from kinking of wires, although 8 surgical interventions were required in this cohort; these included 3 for lead migration (all in 1 patient) and 5 for battery replacement (2 battery depletions in 1 patient). The efficacy in reducing a cluster headache attack was less than deep brain stimulation. In a further open-label study [78] with follow-up postsurgery (mean follow-up, 14.6 months), the mean attack frequency and intensity decreased by 68% and 49%, respectively.

In a French prospective observational study with 1-year follow-up, they evaluated the efficacy of ONS in drug-resistant chronic cluster headache patients as well as the emotional impact [79]. In their study, about 70% of the patients responded to ONS and 40% of the patients were able to decrease their prophylactic treatments.

An open-label study looking into the efficacy of ONS of 35 drug-resistant chronic cluster headache patients, after a median follow-up of 6.1 years (range, 1.6-10.7), showed 66.7% were responders. In this study, they defined responders as 50% reduction in headache attack number per day [80].

The ICON study (NCT01151631) [81] is a prospective, randomized, double-blind, clinical study comparing high-amplitude (100%) and low-amplitude (30%) ONS in medically intractable, chronic cluster headache patients. This study is anticipated to complete in December 2018.

The ONS device has a subcutaneous battery placed in the chest wall or abdomen and leads that are tunneled to join the occipital nerves. From a follow-up study of 35 chronic drug-resistant cluster headache patients, the most common complications were battery depletion (66%) and lead migration (19%) [80]. In a German cost-effectiveness study with ONS for intractable chronic cluster headache and chronic migraine, 25 of 27 patients (93%) responded to treatment. However, 21 complications in 14 patients were identified, necessitating reoperation in 13 cases [82]. With the advancement of ONS stimulation, the Bion microstimulator was developed; this has the benefit of being smaller in size and a transcutaneous rechargeable lithium ion battery [83].

Given the use of occipital nerve injections in cluster headache management, studies have been performed looking into the predictive value of a positive occipital nerve injection prior to ONS insertion. This has not been conclusive, two studies showed no predictive outcome [84, 85], but a recent prospective study found that a previous positive response to a GON injection is associated with a higher likelihood of a positive ONS outcome [86].

The clinical neuromodulation effect from ONS is noticed after months of stimulation, and this is in keeping with the functional neuroimaging findings [87]. Magis and colleagues used FDG–PET to look at the pain matrix, hypothalamus, and brainstem. They found that there were no significant changes between the PET scans when the ONS stimulator was turned on or off within a 72-h delay. Whereas, in the long term (> 6 months), there were reductions in the initial areas of hypermetabolism in chronic cluster headache patients compared to healthy volunteers. The areas of reductions were the anterior cingulate cortex, left pulnivar, left visual cortex, left lower pons, cerebellum, and midbrain. The ipsilateral hypothalamus activation was not reduced, suggesting that the hypothalamic activation is related to the chronic nature of the condition and that ONS is a symptomatic treatment. It should be noted that it is not certain whether the findings from neuroimaging are due to a direct effect from the ONS or from a reduction in frequency of attacks.

Occipital nerve stimulation, including the use of the Bion microstimulator, has been used in cluster headache [76, 77, 80, 88–90]; in addition to this, it has been used in other TACs. In hemicrania continua [75, 90, 91] and more recently in a prospective open-label case series of 16 HC patients, 50% (8/16) of the patients had a favorable response, defined as a more than 50% reduction in monthly moderate to severe headache days [92]. There are 2 case series involving SUNCT/SUNA [93, 94], in the latter, the authors reported 77% of their 31 medically refractory patients had a more than 50% reduction in daily attack frequency with ONS [94]. The use of ONS has thus far only been reported in 1 medically refractory chronic PH case [95]; with follow-up of over 10 years, the patient reported a sustained efficacy of more than 50% reduction in attack frequency and she was able to stop indomethacin completely and was pain free at final follow-up.

### Sphenopalatine Ganglion

The sphenopalatine ganglion (SPG) has an important role in the pathway of TACs and the presentation of parasympathetic features. It has been a historic target for refractory headaches since 1909, when Sluder [96] described treatment of a case series of Merkel’s ganglion neuralgia, where the SPG was targeted using formaldehyde, alcohol injection, or cocaine, as an intranasal spray or as an application of saturated cocaine solution. Later, the SPG was targeted with cocaine in nitroglycerin-triggered cluster headache attacks in 1982 [97], alcohol injection [96, 98], lidocaine injection [99, 100], and radiofrequency ablations, with varying success rates and transient benefit [101].

Comparably, implantation of an SPG stimulator (Pulsante®) in drug-resistant chronic cluster headache has had more sustained results. Following the proof-of-concept study [102], there has been a randomized, blinded, multicenter study, Pathway CH-1 [103], with extended open-label outcome at 24 months [104]. The main findings from these studies were that SPG stimulation can be used for acute treatment of attacks as well as for its neuromodulatory effects. Acute pain relief from SPG stimulation was observed in 55% of patients within 15 min compared to only in 6% and 7% with sham and subthreshold stimulation respectively; this is a net effect that is comparable to that of injectable sumatriptan. In CH-1, there was also an unexpected dramatic reduction in attack frequency noted with repetitive attack stimulation. This therapeutic effect was maintained through the 24-month follow-up, with 45% being acute responders and 33% being frequency responders, and in total, 61% had either or both therapeutic effect [104]. Likewise, the use of both acute and preventive medication was markedly reduced and conversely the quality of life of the patients improved significantly [104]. The main side effects noted from implanting the SPG stimulator were sensory disturbances and pain and swelling in the weeks after the implant procedure. There were 3 patients that required SPG lead revisions and 2 SPG stimulators were removed, so the overall tolerability was favorable [103]. A new large-scale open-label study enrolled 97 medically refractory cluster headache patients (88 chronic and 9 episodic); of the 85 patients who completed the study, they confirmed the initial study results with a response rate of 68% [105]. Thirty-two percent of all patients were acute responders and 55% of the chronic patients were frequency responders [105]. Pathway CH-2 (NCT02168764), is a study based in the USA, assessing the efficacy of SPG as acute treatment in chronic cluster headache. It is estimated to complete in January 2019.

### Vagus Nerve

The vagus nerve is the 10th cranial nerve; it is a mixed sensory and motor nerve with a long course. Vagal nerve stimulation (VNS) has been used as treatment for epilepsy [106] and depression [107] for many years. Two chronic cluster headache patients with coexistent depression had a beneficial response to their cluster headache following VNS implantation; in particular, 1 patient had improvement within 2 months of implantation and there was a reduction in his attack frequency in a year [108].

With technological advancement, a portable and noninvasive vagus nerve stimulation (nVNS) device (gammaCore) was designed and developed. It produces a low-voltage electrical signal comprising a 5-kHz sine wave burst, with 1 stimulation lasting 2 min. The first large pilot open-label single-arm study with nVNS was aimed to investigate its efficacy as an acute treatment for migraine [109]; subsequently, the efficacy of nVNS was studied in cluster headache. In an open-label observational study, nVNS treatment was assessed in 19 cluster headache patients (11 chronic and 8 episodic) [110]. In this study, Nesbitt and colleagues first reported the potential of the nVNS as an acute and preventive therapy for cluster headache patients. There were no serious adverse events from the use of nVNS; the main side effects reported were local discomfort associated with its use and mild skin reactions to the conductive gel. One patient had increase in side-shifting of attacks. Following on from this, the PREVention and Acute Treatment of Chronic Cluster Headache (PREVA) study was the first randomized, prospective, controlled study to investigate nVNS treatment in chronic cluster headache patients, showing that daily use of three 2-min stimulations 5 min apart, administered twice a day, had a beneficial effect on the frequency of attacks per week [111]. After 4 weeks, the weekly attack frequency was 41% lower in the patients receiving prophylactic nVNS and standard of care compared to patients receiving only standard of care [112].

The ACT1 study, a randomized double-blind, sham-controlled prospective study [113], was aimed at assessing the use of nVNS as an acute treatment for cluster headache. The nVNS did provide significant and clinically meaningful benefits over sham treatment in the episodic cluster headache subgroup, providing pain control within 15 min with sustained treatment response. These results were not present in the chronic cluster headache group. The results from the ACT2 study showed that nVNS was superior to sham therapy for acute treatment of attacks in the episodic cluster headache subgroup but not in the chronic cluster headache group or in the total population [114].

The use of nVNS has been explored in other TACs with promising results. Nesbitt and colleagues reported the use in 2 hemicrania continua patients, previously treated with the Bion occipital nerve stimulator. The first patient used the nVNS acutely and prophylactically, with 30% improvement in background pain and 20% improvement in the acute worsenings, aborting the worsenings within 15 min. In the second patient, the device was used purely as a prophylactic treatment; there was a 75% improvement in the background pain and acute worsenings [115]. A hemicrania continua patient intolerant of indomethacin reported beneficial effects from nVNS; it consistently reduced the level of pain during exacerbations from 8/10 to 5/10 in severity [116]. In a clinical audit of nVNS use in indomethacin-sensitive headaches, 9 patients had hemicrania continua and 6 had paroxysmal hemicrania; the authors reported a 78% improvement in HC patients in their baseline pain, and 67% of PH patients reported a reduction in both attack frequency and severity [117]. nVNS is a particularly helpful alternative for HC and PH patients who are intolerant of indomethacin.

## Molecular Targets

### Calcitonin Gene-Related Peptide

Calcitonin gene-related peptide is a 37-amino-acid protein that comes in 2 forms, α and β isoforms. It is found in trigeminal afferents and plays a key role in the trigeminal–autonomic reflex. This is supported by the key study by Goadsby and colleagues [118], whereby they showed there was an increase in plasma calcitonin gene-related peptide (CGRP) levels when the trigeminal ganglion was electrically stimulated. Furthermore, in vivo studies measuring plasma CGRP from the external jugular vein showed it is elevated during cluster headache attacks, both during spontaneous [119] and nitroglycerin-triggered attacks [120, 121]. With the plasma CGRP levels normalizing after treatment of attacks with sumatriptan or oxygen, the normalization did not occur if the attacks were treated with opioids [119], implying attenuation of neuropeptides plays an important role in treatment of attack.

There are currently 4 randomized control trials investigating the use of CGRP monoclonal antibodies in cluster headaches, Galcanezumab (LY2951742) and Fremanezumab (TEV-48125), in participants with episodic cluster headache and chronic cluster headache. Furthermore, CGRP has been used to trigger cluster headache attacks successfully in chronic and episodic cluster headache patients “in bout,” but not in patients “outside bout.” These results are from a late-breaking abstract [122] and may cautiously suggest efficacy of CGRP antagonists in cluster headache treatment.

At present, the CGRP monoclonal antibodies have not yet been tried in the other TACs; however, it may be useful in CPH. The role of CGRP in CPH is not clear; however, similar to cluster headache, it has been noted that the plasma CGRP level and VIP rises during a CPH attack and normalizes following treatment with indomethacin [123].

### Somatostatin

Somatostatin is an endogenous 14-amino-acid peptide, which plays a pivotal role in the regulation of the neuroendocrine system and in pain modulation. Somatostatin receptors are found throughout the brain, including sites that are involved in nociceptive processing and the hypothalamus [124]. This is supported by an animal study, whereby the authors showed that blocking somatostatin receptors in the posterior hypothalamus with cyclosomatostatin caused an antinociceptive effect on dural and facial input, while injection of somatostatin to the posterior hypothalamus did not have an effect on either dural or facial afferent input to trigeminal neurons [125]. Furthermore, somatostatin is involved in modulating neuropeptides; in particular, somatostatin can reduce CGRP levels [126]. In cluster headache patients, it has been shown that they have lower levels of plasma somatostatin during both attacks and attack-free periods, when compared to normal healthy individuals [127].

A double-blind study aimed at assessing the efficacy of intravenous somatostatin infusion (25 μg/min for 20 min) in the acute treatment of cluster headache attacks, when compared with treatment with intramuscular ergotamine (250 μg) and with placebo [128], showed that somatostatin reduced the maximal pain intensity significantly compared with placebo and almost comparable to ergotamine. However, logistically, it is not practical for patients to use intravenous infusion as acute treatment for recurrent and multiple daily attacks. Therefore, in a further study, subcutaneous somatostatin was compared with ergotamine and it was shown that it was as beneficial as ergotamine in reducing pain but less effective in reducing the duration of cluster headache attacks [129]. This may be explained by the short half-life of somatostatin, whereas somatostatin analogues such as octreotide have a longer half-life of 1.5 h [130]. A randomized placebo-controlled double-blind crossover study, using subcutaneous octreotide in acute treatment of cluster headache, showed that in 52% of the attacks treated with octreotide, patients became headache free at 30 min compared with 36% of the attacks treated by placebo [131]. A multicenter, placebo-controlled, single-dose study is underway to assess the safety and effectiveness of pasireotide (SOM230), a long-acting release somatostatin analogue, in acute cluster headache treatment (NCT02619617).

### Transient Receptor Potential Vanilloid Receptor

Transient receptor potential vanilloid (TRPV-1) receptors play a pivotal role in nociception and in particular explained the mechanism of action of plant-derived analgesics, such as capsaicin [132]. Civamide is a synthetic isomer of capsaicin, a TRPV-1 receptor modulator, and it can selectively depress activity in type-C nociceptive fibers and causes a release and subsequent depletion of neuropeptides, including substance P and CGRP [133]. Civamide is not systemically absorbed; the benefit of this treatment is that it does not produce systemic adverse side effects and does not interact with other medications for cluster headache or concomitant medical problems. Side effects of civamide include a localized burning sensation at the application site [134].

In 2002, a multicenter, double-blind, randomized, vehicle-controlled study with a 7-day treatment period in 28 episodic cluster headache patients was performed with a 20-day follow-up comparing 100 μL of 0.025% civamide (25 μg) in each nostril once a day with 100 μL of vehicle. They found that civamide was modestly effective in the preventive treatment of cluster headache and reduced the frequency of cluster headache attacks [133]. In a larger unpublished study of 112 patients, civamide decreased the frequency of cluster headache attack per week; however, this was not statistically significant [134]. A phase III multicenter, double-blind, randomized, vehicle-controlled study with 0.01% civamide nasal solution (NCT01341548) is due to start in November 2018, with the aim to evaluate the safety and efficacy of intranasal civamide solution in episodic cluster headache periods.

### Nitric Oxide

Nitric oxide (NO) plays an important role in headaches, particularly in migraine and cluster headache [135, 136]. NO is involved in regulating cerebral and extracerebral blood flow and arterial diameter; however, it is also involved in nociceptive processing. Several studies have been performed to investigate the role of NO in cluster headache pathogenesis by measuring its plasma end products, showing enhanced plasma nitrite levels in cluster headache patients in and out of bout [137] and higher levels of NO oxidation and end products in the cerebrospinal fluid in cluster headache patients in and out of bout, when compared with healthy volunteers [138]. Other studies did not confirm this difference; in particular, Costa and colleagues found that the baseline plasma NO end metabolite, nitrite level, and l-citrulline levels between cluster headache patients and healthy controls were similar and showed that plasma nitrite levels during nitroglycerin-triggered cluster headache attacks during peak pain were increased, but this increase was also increased in the healthy controls 45 min after nitroglycerin [139]. Although the results from plasma NO end metabolites studies have not been conclusive, nitroglycerin, a prodrug for nitric oxide, has been used as a systematic method to trigger cluster headache attacks since it was described by Ekbom in 1968 [140].

Paroxysmal hemicrania and hemicrania continua are both indomethacin-sensitive headaches. The mechanism of indomethacin is not clear, and although it is classified as a nonsteroidal anti-inflammatory drug (NSAIDs), it has properties that set it apart from the rest of the NSAIDs. From animal studies, indomethacin seems to inhibit the NO-related mechanism, setting it apart from the other NSAIDs that were investigated in the study, naproxen and ibuprofen [141]. To further support the role of NO in PH pathophysiology, nitroglycerin has been reported to trigger PH attacks [142].

Therefore, the NO-cGMP cascade offers opportunities as a potential therapeutic target for TAC treatment. Moncada and Higgs [143] have described the NO therapeutic targets throughout the whole NO cascade in their detailed review. The results from NO synthase (NOS) inhibition in migraine have not been encouraging. The first clinical trial using a non-selective NOS inhibitor L-N^G^methylarginine hydrochloride (L-NMMA, 546 C88) to assess its efficacy in acute migraine treatment [144] showed the headache response at 2 h was 67% in the treatment arm compared with 14% in the placebo arm. A highly selective inducible NOS (iNOS) inhibitor (GW274150) was studied as migraine acute treatment [145] and preventive treatment [146, 147], but the studies did not show a statistically significant treatment advantage over placebo.

From the evidence in support of NO in TAC pathogenesis, the NO–cGMP cascade should be a suitable therapeutic target.

### Melatonin

Melatonin is an indole compound produced by the pineal gland and is secreted when in darkness and suppressed by light; this process involves the suprachiasmatic nucleus of the hypothalamus [148]. Melatonin plays an important role in regulating circadian rhythms, including sleep initiation and sustaining sleep. There have been several studies showing abnormal melatonin levels in cluster headache. In patients with episodic cluster headaches, there is blunting of the nocturnal peak of melatonin [12–15, 38–40]. From these studies, the role of exogenous melatonin supplementation has been studied as treatment options for cluster headache. In a randomized placebo-controlled trial, 10 mg melatonin daily orally for a fortnight was tried in predominantly episodic cluster headache patients; from a total of 20 patients, 18 had episodic cluster headache and 2 had chronic cluster headache [149]. They showed that 5 out of 10 patients in the melatonin arm responded to melatonin; the 2 patients with chronic cluster headache did not respond. A case report of 2 chronic cluster headache patients responded to melatonin supplementation 9 mg [150]. A study with the aim of assessing the benefits of adjunctive treatment using 2 mg controlled-release melatonin in cluster headache management in both chronic and episodic cluster headache patients did not show there was a therapeutic benefit of the additional melatonin in both chronic and episodic cluster headache patients that were uncontrolled on conventional therapy [151]. However, as melatonin is associated with minor or no side effects, it may be added to existing therapy and it may still be helpful to start melatonin earlier in the bout as suggested by Leone and colleagues [149].

Melatonin has a shared indole structure with indomethacin; therefore, the therapeutic effect of melatonin has been tried in indomethacin-sensitive headache, hemicrania continua in particular. A small case report of 3 patients did respond to melatonin dose, 2 patients responded to 9 mg melatonin, and 1 patient required 15 mg melatonin to reduce her indomethacin dose from 150 mg daily to 75 mg daily [152]. A subsequent study used melatonin as a preventive therapy in 11 patients with hemicrania continua, starting with 3 mg melatonin for 5 nights, increasing to 6 mg for 5 nights, and then 9 mg for at least 5 nights. Unfortunately, only 20% achieved pain freedom with melatonin; however, 45% of patients had a complete or partial response and thereby could reduce their daily dose of indomethacin [153].

### Orexin

Orexin A and orexin B are integral to sleep and feeding, but also play a role in nociception and can modulate the autonomic nervous system. Therefore, orexin is thought to play a role in cluster headache and a lower CSF concentration is also demonstrated in cluster patients than in healthy controls [154]. Orexins are synthesized in the lateral and posterior hypothalamus and project widely to nociceptive areas of the brain and spinal cord, and receptor activation can modulate responses of the trigeminovascular system to dural stimulation [42]. An exploratory phase II study using Filorexant (MK-6096), an orexin receptor 1 and 2 antagonist, has been tested in migraine prevention; however, it was not found to be effective [155]. Currently, we are not aware of any studies in cluster headache.

### Pituitary Adenylate Cyclase-Activating Polypeptide 38

PACAP-38 is a 38-amino-acid neuropeptide [156] found in the sphenopalatine ganglion, the otic ganglion, and trigeminal ganglion [157] and is structurally related to vasoactive intestinal polypeptide (VIP). PACAP-38 is released in acute cluster headache attacks, and episodic cluster headache patients, when out of bout, have low plasma PACAP-38 levels compared with healthy controls [158]. Furthermore, it plays a role in the circadian rhythm and can modulate melatonin synthesis. From animal studies, in the suprachiasmatic nucleus, PACAP-38 can cause a phase shift in the circadian rhythm via a cAMP signaling pathway [159].

In humans, PACAP-38 has been used to bring on migraine-like headache in patients with migraine without aura [160, 161] by means of PAC1 receptor activation, indicating the PAC1 receptor as a potential future treatment target [162]. PACAP-38 has not yet been used to trigger cluster headache attacks; however, it would be interesting to assess its role in the pathogenesis of cluster headache attacks [163] and could be a future therapeutic target.

AMG301 (PAC1 antibody) has been developed and is undergoing a phase IIa, randomized, double-blind, placebo-controlled study in chronic or episodic migraine (NCT03238781), and the study is estimated to complete in December 2018. Depending on the results, this may also be promising in cluster headache.

### Glutamate

Glutamate is an excitatory neurotransmitter that acts on ionotropic glutamate receptors that can be divided into 3 groups: N-methyl-d-aspartate (NMDA), α-amino-3-hydroxy-5-methyl-4-isoazolepropionic acid (AMPA), and kainate. Glutamate plays a key role in the induction of nociceptive sensitization and in the trigeminovascular pathway [164]. To support this, memantine, a NMDA-gated ion channel blocker, has shown efficacy in reducing CH attacks in resistant patients [165]. This may suggest changes to the NMDA receptor signaling or NMDA receptor activation may be relevant to the pathophysiology of CH. Further, to support the role of glutamate in cluster headache pathophysiology, a study investigated the serum levels of kynurenine metabolites in cluster headache [166]. The kynurenine metabolic pathway of tryptophan generates neuroactive metabolites that influence the activity of NMDA receptors as well as other glutamate receptor types. The study found that cluster headache patients have altered kynurenine metabolites when compared with healthy controls and, in particular, reduced levels of kynurenic acid (an NMDA receptor antagonist) support the hypothesis that NMDA receptors are overactive in CH and a potential therapeutic target.

## Conclusion

The therapeutic targets for TAC treatment have greatly expanded over the years with our accumulating understanding of the pathogenesis of TACs. Conventional TAC management was limited and involved medications that have been borrowed from other conditions and are often hampered by intolerable side effects. This is an exciting era in TAC therapy, with multiple and diverse therapeutic targets for better and more effective treatment options. We will be able to expand the therapeutic armentarium and move away from the most invasive treatment options to more condition-specific, targeted, and noninvasive treatments, treatments developed with TACs in mind.

## Electronic Supplementary Material

ESM 1 (PDF 499 kb)

## Abbreviations

GON: Greater occipital nerve
VNS: Vagal nerve stimulation
nVNS: Noninvasive vagus nerve stimulation
TAC: Trigeminal autonomic cephalalgia
PH: Paroxysmal hemicrania
HC: Hemicrania continua
SUNCT: Short-lasting unilateral neuralgiform headache attacks with conjunctival injection and tearing
SUNA: Short-lasting unilateral neuralgiform headache attacks with cranial autonomic symptoms
SSN: Superior salivatory nucleus
VIP: Vasoactive intestinal polypeptide
DBS: Deep brain stimulation
SPG: Sphenopalatine ganglion
CGRP: Calcitonin gene-related peptide
NO: Nitric oxide
PACAP: Pituitary adenylate cyclase polypeptide
TCC: Trigeminocervical complex
ONS: Occipital nerve stimulator
SCN: Suprachiasmatic nucleus
NMDA: N-Methyl-d-aspartate
AMPA: α-Amino-3-hydroxy-5-methyl-4-isoazolepropionic acid
TRPV: Transient receptor potential vanilloid
CSF: Cerebrospinal fluid
NOS: Nitric oxide synthase
cGMP: Cyclic guanosine monophosphate
cAMP: Cyclic adenosine monophosphate
